# Mouse Models of Hepatocellular Carcinoma: Classification, Advancement, and Application

**DOI:** 10.3389/fonc.2022.902820

**Published:** 2022-06-30

**Authors:** Sha Liu, Fang Huang, Guoqing Ru, Yigang Wang, Bixiang Zhang, Xiaoping Chen, Liang Chu

**Affiliations:** ^1^ Hepatic Surgery Center, Tongji Hospital, Tongji Medical College, Huazhong University of Science and Technology, Wuhan, China; ^2^ Cancer Center, Department of Pathology, Zhejiang Provincial People’s Hospital, Affiliated People’s Hospital, Hangzhou Medical College, Hangzhou, China; ^3^ College of Life Sciences and Medicine, Zhejiang Sci-Tech University, Hangzhou, China

**Keywords:** hepatocellular carcinoma, mouse model, gene-edited mice, metastasis, transplantation model

## Abstract

Hepatocellular carcinoma (HCC) is the subtype of liver cancer with the highest incidence, which is a heterogeneous malignancy with increasing incidence rate and high mortality. For ethical reasons, it is essential to validate medical clinical trials for HCC in animal models before further consideration on humans. Therefore, appropriate models for the study of the pathogenesis of the disease and related treatment methods are necessary. For tumor research, mouse models are the most commonly used and effective *in vivo* model, which is closer to the real-life environment, and the repeated experiments performed on it are closer to the real situation. Several mouse models of HCC have been developed with different mouse strains, cell lines, tumor sites, and tumor formation methods. In this review, we mainly introduce some mouse HCC models, including induced model, gene-edited model, HCC transplantation model, and other mouse HCC models, and discuss how to choose the appropriate model according to the purpose of the experiments.

## Introduction

There are two major types of primary liver cancer, hepatocellular carcinoma (HCC) and intrahepatic cholangiocarcinoma (iCCA). HCC is the most common type and accounts for more than 90% of cases, and the other, iCCA, is about 10%–15% (1). The incidence rate of HCC is higher in people with long-term liver diseases, such as cirrhosis caused by hepatitis B virus (HBV) or hepatitis C virus (HCV) infection (2). People with non-alcoholic steatohepatitis (NASH), associated with metabolic syndrome or diabetes mellitus, are also considered to be at high risk for HCC incidence, particularly in the West (3). The processing of HCC has undergone multiple stages such as initiation, promotion, and evolution and is closely related to the regulation and expression of genes (4). At present, the pathogenesis of HCC has not been clarified totally. No effective treatment has been found for advanced HCC (5, 6). Although epidemiological studies have shown that HBV and HCV infection, aflatoxin, alcohol, nitrosamines, and other substances are related to the incidence of HCC (7, 8), the molecular mechanism and pathway of HCC are still unclear (9). Building a more suitable model for human HCC research is helpful to solve these problems.

The models can be roughly divided into two types, *in vitro* models and *in vivo* models. *In vitro* (Latin for “in the glass”) studies are performed with microorganisms, cells, or biological molecules outside their normal biological context. These experiments can explore the problem from more detailed molecular mechanisms and more single influencing factors, but the disadvantage is that the conditions are too simple to mimic the extremely complicated living environment in the body (10). *In vivo* (Latin for “within the living”) studies are those in which the effects of various biological entities are tested on whole living organisms, usually animals. The advantage of this model is closer to the real-life environment, so the repeated results on this model are closer to the real situation (11, 12). To clarify the relationship between risk factors and the development of HCC, a large number of researchers have applied HCC animal models, and the methods used to establish these models are varying.

The conservation of mouse genetics is very similar to humans; on the other hand, the technology of gene editing becomes easier and more economic. All of these make a mouse model one of the most important tools for the study of the biological characteristics of HCC and screening of new drugs. The formation of liver cancer is an extremely complex process. There are many similarities between the construction methods of HCC and iCCA mouse models. Several mouse models reflect the full spectrum of liver cancers, from HCC to iCCA and to mixed tumors. In the present review, we mainly introduce the mouse HCC models, including induced model, gene-edited model, HCC transplantation model, and other mouse HCC models (**Table 1**), and discuss the selection of appropriate mouse HCC models for different experimental needs.

**Table 1:** Comparison of different types of mouse HCC models.

Model type	Choice of mice	Advantage	Disadvantage	Mouse age
Induced mouse HCC models	C3H mice, C57BL/6 mice, B6C3F1 mice	Stable, mimic the natural state	Uneven growth, experiment cycle is long	7–15 days
Gene-edited mouse HCC models	All kinds of mice	Mimic the genetic deletion patients	Tumors are multiple and scattered in the liver, some other unexpected defection in different tissues	Embryo
HCC transplantation mouse models	Nude mice, SCID mice, NOD-SCID mice for *Homo sapiens* resource cell. Others for murine cell.	Uniform, *Homo sapiens* resource, easy and fast	Hard to observe (*in situ*), limited to cell lines and mouse strains	4–8 weeks
HBV-infected mouse models	Immunodeficient mice	Mimic virus-induced HCC	Hard to promote viral infection	Embryo
HCC metastasis model	Immunodeficient mice	Mimic metastasis	Not stable and non-mature	4–8 weeks

HCC, hepatocellular carcinoma; HBV, hepatitis B virus; SCID, severe combined immunodeficiency; NOD-SCID mice, non-obese diabetic severe combined immunodeficient mice.

## Chemically Induced Mouse Hepatocellular Carcinoma Models

**Figure 1:**
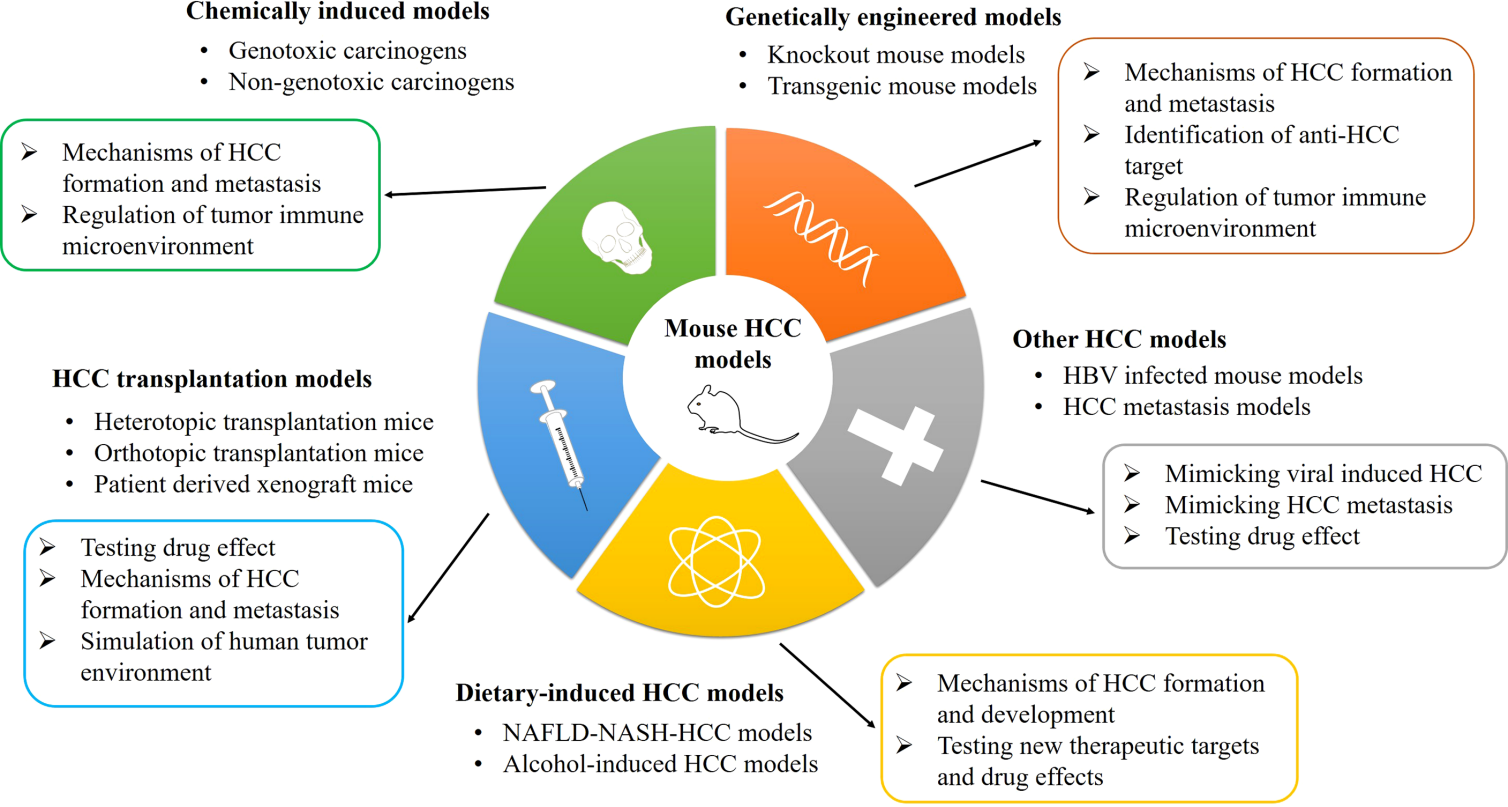
The classification, advancement, and application of mouse HCC models. HCC, hepatocellular carcinoma; HBV, hepatitis B virus; NAFLD-NASH, non-alcoholic fatty liver disease-non-alcoholic steatohepatitis.

Chemical drug induction is a very stable liver cancer modeling method, and it is the best way to restore the true state of liver cancer in mice. In these models, three processes of tumorigenesis were simulated, namely, injury, sclerosis, and tumor (13). The substances that cause liver cancer could be separated into two main categories: genotoxic carcinogens and non-genotoxic carcinogens (14). Genotoxic carcinogens are chemical carcinogens that can react with DNA and cause DNA damage, such as aflatoxin B1 (AFB1), ethidium bromide (EB), and diethylnitrosamine (DEN) (15). Non-genotoxic carcinogens do not react directly with DNA, such as carbon tetrachloride (CCl_4_) and alcohol. A lot of chemical carcinogens need sufficient doses and time to induce tumor formation in animals. The generation of tumor is almost caused by inducing certain key lesions in host cells, such as controlling cell proliferation, apoptosis, and differentiation (16). Therefore, carcinogen is the first choice to induce HCC in mice to mimic the progression in human beings, and the most commonly used inducers include DEN, AFB1, and CCl_4_ (17, 18). The classification, advancement, and application of various mouse HCC models are shown in **Figure 1**.

### Genotoxic Carcinogens

DEN induces not only liver cancer, but also gastric cancer, skin cancer, and blood tumors. DEN can cause DNA alkylation damage, leading to tumorigenesis. Studies have shown that the occurrence of DEN-induced liver cancer is related not only to the injection dose and time but also to the strain, gender, and age of mice (19). AFB1 is a secondary metabolite produced by *Aspergillus flavus*, *Aspergillus parasiticus*, and other strains. AFB1 mainly causes DNA chemical damage by changing the structure of DNA, thereby inducing the formation of liver cancer (20).

### Non-Genotoxic Carcinogens

The hepatotoxicity of CCl_4_ is mainly divided into two stages. First, free radicals are metabolized in the liver. Free radicals can cause damage to cell membranes. Second, they can cause inflammatory response reactions and lead to the secretion of inflammatory factors, chemokines, and other pro-inflammatory factors that directly damage the liver. Such repeated damage, inflammation, and repair will eventually cause liver fibrosis and liver cancer (21).

A study group has attempted to use prototypic non-genotoxic carcinogens thioacetamide (TAA) and methapyrilene (MP) to induce hepatocarcinogenesis in rats. Their results have shown that repeated treatment with chemical compounds led to similar expression patterns of signature genes based on a toxicogenomics approach (22). They further found that cumulative treatment using non-genotoxic TAA might play an initiating potential in hepatocarcinogenesis (23). This implies for its application in mouse HCC models.

There are still some disadvantages for this method such as long induction period and high lethality. Although chemical drug induction can be used to establish HCC models, the homogeneity of tumorigenesis will be affected by multiple factors, including mouse gender, age, strain, genetic background, and so on (24), which make the tumor progression in different mice not uniform. Therefore, if researchers want to study a specific gene and its effect on progression of liver cancer, a gene-edited mouse model might be more suitable.

### Methodology of Chemical Carcinogen-Induced Hepatocellular Carcinoma

The chemical carcinogens could induce hepatocarcinogenesis *via* different doses and time in mouse models. As a genotoxic carcinogen, DEN treatment alone effectively caused HCC in mice. Based on previous studies, the used dose of DEN varied from 1.25 to 100 g per kg body weight of mouse *via* intraperitoneal (i.p.) injection (19, 25). According to the nontoxic dose, the formation time of HCC will be 6 months later, and final HCC incidence is time-dependent and can reach 100% (26). In addition, the formation time and incidence of HCC are also dose-dependent. Further study found that hepatocarcinogenesis induced by DEN is closely associated with mouse gender, age, and genetic background (19). The incidence of DEN-induced HCC is higher in male mice, which is similar to that of human with high HCC occurrence in men. It was also proven that the DEN-induced HCC in male mice is reduced by orchiectomy or inhibition of gonadotropin (27). For mouse age prone to develop liver tumor, the best age range is from seventh to 15th day because newborn mice at this period have better enzyme activity to hydroxylate DEN (28). Moreover, among the commonly used mouse strains, C3H mice are the most sensitive to DEN-induced HCC where the HCC incidence attained 30%–50% than that of 20%–30% in C57BL/6 × C3H F1 mice (B6C3F1 mice) and <2.5% in C57BL/6 mice (19, 29). Another study tested the two-stage progress from fibrosis to HCC *via* i.p. injection of DEN following CCl_4_ administration (16, 30). In this model, the mouse strain used is B6C3F1 mice injected with 1 mg/kg mouse body weight of single DEN at the 14th day of age and 0.2 ml/kg of i.p. CCl_4_ 2 times per week starting at the eighth week of age up to the 14th week (16). The result showed that the treatment led to 100% incidence of liver tumor adenomas expected at 5 months of age (30).

## Diet-Induced Mouse Hepatocellular Carcinoma Models

Currently, more and more diseases are closely related to diet and lifestyles. The increasing HCC rates might be partially attributed to non-alcoholic fatty liver disease (NAFLD) and NASH (31). Thus, it is necessary to clearly clarify the molecular mechanisms of liver specificity and to establish mouse models for the NAFLD-NASH-HCC progression. A group has suggested a dietary NASH mouse model using a choline-deficient, L-amino acid-defined, high-fat diet (CDAHFD) and found that CDAHFD-fed C57BL/6J mice developed chronic advanced hepatic fibrosis and further fibrosis-associated autochthonous HCC with the features of trabecular, pseudoglandular, and solid growth (32). Therefore, the CDAHFD mouse model provides a useful tool for studying pathological changes of hepatocarcinogenesis from NAFLD/NASH.

Asgharpour et al. (33) has reported a diet-induced mouse model of NAFLD and HCC in a cross between 129S1/SvImJ and C57Bl/6J mice using a high-fat diet with *ad libitum* glucose and fructose in physiological concentrations and found that this mouse model can mimic the physiological, metabolic, histological, and transcriptomic changes of human progressive NASH and HCC. Another study also established a NASH mouse model using a western diet with high fat, fructose, and cholesterol combined with a low weekly dose of i.p. CCl_4_, which resulted in rapid progression of advanced fibrosis and HCC similar to the features of human NASH (34). These diet-induced NAFLD-NASH-HCC models are easily reproducible and can facilitate to test new therapeutic targets for NASH in preclinical trials.

It is worth noting that the increasing incidence of NASH-driven HCC could be promoted by some factors. It included the combination of long-term liver X receptor agonist (T0901317) stimulation with oxidative stress and a high-fat diet (35), and steroidogenic acute regulatory protein 1 (STARD1) stimulated the generation of bile acid in the mitochondrial acidic pathway (36). Furthermore, in mouse models of NAFLD, dysregulation of lipid metabolism causes a selective intrahepatic CD4^+^ but not CD8^+^ T lymphocyte loss and further accelerates hepatocarcinogenesis, which is consistent to that of human samples (37). Due to higher mitochondrion-derived reactive oxygen species (ROS) in CD4^+^ than CD8^+^ T lymphocytes, the *in vivo* blockade of ROS reversed NAFLD-induced CD4^+^ T-lymphocyte depletion and retarded NAFLD-induced HCC (37). This study implies that adaptive immunity plays an important role in NAFLD-promoted HCC.

Besides NAFLD/NASH-related HCC models, mouse models for alcohol abuse are increasing research hot spots because chronic alcohol consumption is a crucial risk factor for hepatocarcinogenesis (38). In a mouse model of alcohol-induced HCC, it was found that (Interleukin 17A) IL-17A plays a critical tumor-promoting role in regulating inflammatory responses in macrophages and cholesterol synthesis in steatotic hepatocytes, which suggests that it may be a potential target for the treatment of patients with alcohol-induced HCC (39).

In patients and mouse models, another important study has reported that aldehyde dehydrogenase 2 (ALDH2) loss accelerates alcohol-induced HCC development (40). Mechanistically, abundant harmful oxidized mitochondrial DNA *via* extracellular vesicles is produced by *aldh2*-deficient hepatocytes after chronic alcohol exposure, which can activate multiple oncogenic pathways facilitating HCC development (40). Moreover, chronic alcohol exposure is related to HCC stemness and metastasis through TLR4-NANOG pathway-dependent cancer stem cells (41) and β-catenin/miR-22-3p/TET2 axis (42). Reverse study proved that obesity, but not alcohol, promotes HCC incidence and progression and increases HCC number and size in a mouse model independent of chronic alcohol consumption (43). These studies enhanced the complexity of HCC formation and development and the importance of animal models of HCC.

## Genetically Engineered Mouse Models

Researchers studying mice constructed by genetic engineering can not only conduct research at the level of tissues and organs but also penetrate down to the level of cells and molecules, which can provide ideal experimental animal models for the pathogenesis of cancer, drug screening, and clinical medical research (44). In the field of gene editing, zinc finger nucleases (ZFNs), transcription activator-like effector nucleases (TALENs), clustered regularly interspaced short palindromic repeats (CRISPR)/CRISPR-associated endonuclease cas9 (Cas9), and other methods were invented in the last decades (45) that make it possible and easier to construct gene-edited mouse liver cancer models (46).

### Knockout Mouse Models

Adenomatous polyposis coli (*APC*) is an antagonist of Wnt/β-catenin signaling (47). As a member of the tumor suppressor gene family, APC plays a key role in tumorigenesis and Wnt/β-catenin activation. Colnot et al. (48) added a loxP sequence to both sides of the exon 14 of the mutant APC allele to establish the Apc^lox/lox^ mouse strain by genetic modification. Then, an adenovirus-mediated system was used to specifically deliver Cre recombinase to the liver, and the *APC gene* was invalidated in the liver. In this APC liver-specific knockout model, 67% of mice developed significant liver tumors within 8–9 months after Cre recombinase treatment (48). Except *APC*, other models such as Acyl-CoA oxidase (*Aox*) gene deletion mouse model generated by homologous recombination in embryonic stem (ES) cells can exhibit severe fatty liver, and eventually, it will lead to sporadic cell death, steatohepatitis, lipoma, and cancer (49, 50). *Mdr2*-knockout mouse models by homologous murine *mdr2* targeting in the mouse ES cells have also been reported (51). The *Mdr2 gene*, also known as *Abcb*4, encodes a membrane-bound phospholipid-flipping enzyme that helps phospholipids enter the bile (52). It helps to dissolve cholesterol and inactivate the ionic detergent activity of bile salts. Therefore, when Mdr2 gene is deleted, the concentration of phospholipids in the bile ducts will be reduced (53). The lack of bile components of phospholipids may cause bile ducts to be damaged, precipitate gallstones, induce inflammation, and further lead to liver cancer (54). Studies have shown that *Mdr2*-knockout mice can develop phenotypes of hepatocyte damage, vasodilation, and duct hyperplasia in 2–3 weeks after birth. After 8–9 weeks, mice developed symptoms of liver fibrosis for 16 months. Later, most mice displayed liver cancer lesions. The model lacks liver-specific P-glycoprotein, which can transport lecithin across membranes to the bile duct membranes, eventually leading to inflammation-induced HCC (55). Another model is about transforming growth factor β-activated kinase 1 (*Tak1*) gene, which is a member of the MAP3K family. TAK1 can be activated by cytokines such as TGF-β, interleukin-1 (IL-1), T-cell receptor (TCR), B-cell receptor (BCR), and ceramide (56). Activated TAK1 participates in many important physiological and pathological processes through mediating a series of signal pathways, such as cell proliferation, apoptosis, natural immunity, and acquired immune response. Studies showed that hepatocyte-specific knockout of the *Tak1 gene*, generated by crossing Tak1^flox/flox^ mice with Albumin-Cre Tg mice, can induce apoptosis and necrosis of liver cells 4 weeks after birth and induce primary liver tumors after 4 months. These tumors are histologically and genetically similar to human liver cancer (57, 58).

The abnormal expression of miRNAs has been confirmed to be specifically related to the occurrence of liver cancer. Various miRNA gene-modified liver cancer models have been established. For example, both miR-122 constitutive (whole-body) knockout and liver-specific knockout can establish a stable liver cancer model (59, 60). miR-122 constitutive knockout mice began to develop inflammation at 5 weeks of age, and 89% of male mice and 23% of female mice developed liver tumors at 10 months of age. miR-122 liver-specific knockout mice began to show inflammation at 8–10 weeks of age, and 50% male mice and 10% female mice developed liver tumors at 12 months of age (61).

There are also some genetically engineered mouse models of iCCA, such as liver-specific targeted disruption of tumor suppressors *Smad4* and *Pten* (Alb-Cre/Smad4^loxP/floxP^/Pten^loxP/floxP^) (62), tissue-specific activation of *Kras* and deletion of *p53* (Alb-Cre/loxP-stop-loxP-Kras^G12D^/p53^loxP/loxP^) (63), and liver-specific *Kras* activation and *Pten* deletion (Alb-Cre/Pten^loxP/loxP^/loxP-stop-loxP-Kras^G12D^) (64, 65). Last year, Di-Luoffo et al. (66) generated an experimental mouse model of iCCA that combines cholangiocyte-specific expression of Kras^G12D^ with 3,5-diethoxycarbonyl-1,4-dihydrocollidine (DDC) diet-induced inflammation. Briefly, mice expressing tamoxifen-inducible Cre^ER^ recombinase specifically in cholangiocytes (Osteopontin-iCreERT^2^) were mated with LSL-Kras^G12D/+^ mice, which contain an inducible mutant Kras^G12D^ allele. In this study, 6-week-old mice were administered i.p. tamoxifen three times at a 2-day interval to induce Kras^G12D^ expression followed by 4 weeks of a normal diet. Thereafter, chronic cholangitis was induced by feeding the mice in a 2-week DDC/1-week normal diet/31-week DDC diet manner. Mice expressing Kras^G12D^ in cholangiocytes and fed a DDC diet developed cholangitis, ductular proliferations, intraductal papillary neoplasms of bile ducts (IPNBs), and, eventually, iCCAs.

### Transgenic Mouse Models

In addition to knockout mice, gene transgenic mice can also be constructed using gene editing. The basic method is to transfer the overexpressed gene sequence under the control of liver specifically expressed promoter into the mouse embryo. Dubois et al. (67) constructed an antithrombin-III regulatory sequence-promoted simian virus 40 T-antigen (SV40 T-Ag) expression mouse model. In the mouse strain with the highest expression level of large T-Ag, all of them were detected in the liver tumor when mice had grown to 8 months of age, and 10% of them had metastasis. After that, more and more teams are using the strategy of conditionally initiating proto-oncogene mutation to construct a mouse liver cancer model. For example, a kind of mouse model that can co-overexpress *c-myc* and *TGF-α* in liver (Alb-c-myc/MT-TGF-α) was constructed. It was confirmed that the double transgenic mouse model manifested the occurrence of liver cancer in 100% of males and 30% of females at 8 months of age, and the double transgenic mice developed liver cancer earlier than the mice that overexpressed *c-myc* or *TGF-α* alone. This method can well simulate the increase of carcinogenicity caused by genetic factors such as genetic mutations in clinical practice and is very close to the natural state (68, 69).

In addition to gene editing at the embryonic stage, there are many other ways to construct mouse liver cancer models. For the most common way, transposable elements and hydrodynamic tail vein injection (HTVI) is a useful technology to build a liver cancer model, which can deliver nucleic acids into living mice (70). p19Arf^-/-^ mice, with the loss of the important tumor suppressor gene, can be used as the model for plasmid with transposable element hepatic delivery *via* the HTVI technique. These mice have a high incidence of tumors. Seehawer et al. (71) engineered transposon vectors coexpressing oncogenic mouse *Myc* and human *NRAS* or mouse *Akt1*, which can upregulate the expression of MYC to induce the Mitogen-activated protein kinase-extracellular signal-regulated protein kinase (MEK-ERK) and Phosphatidylinositol 3-kinase-mammalian target of rapamycin (PI3K-mTOR) signaling. Experiments showed that this method can stably produce a mouse liver cancer model (71). In addition to the abovementioned combinations, there are some other combinations such as *APC* and *β-catenin*, *PTEN* and *p53*.

HTVI technology was also used in the construction of iCCA models. Activated forms of AKT (myristoylated AKT1, 20 μg) and an unphosphorylatable form of Yap (YapS127A, 30 μg) proto-oncogenes were injected into FVB/N mice. As early as 3 weeks after injection, tumor lesions with a ductular phenotype were observed (72). A constitutively active human *NRAS* oncogene (G12V NRAS, pT/Caggs-V12Nras, 25 µg) was injected into C57BL/6J p19Arf-null mice, and mice developed mixed HCC/iCCA 4–6 weeks after injection (73).

The HTVI and the CRISPR-Cas9 technology could be combined for the study of tumors. Single guide RNAs (sgRNAs) targeting *p53* and *Pten* were injected into wild-type FVB mice, and 3 months post-injection, all 5 mice coinjected with sgPten and sgp53 developed liver tumors with bile duct differentiation features, recapitulating the liver lesions caused by Cre-loxP-mediated deletion of *Pten* and *p53* (74). Another work indicated that a pool of 10 sgRNAs was injected with a Cre recombinase transgene into Alb-Cre/Kras^LSL-G12D/+^ mice, causing the formation of HCC and iCCA histology 20–30 weeks post-injection (75).

All of these studies have shown a blueprint to us that genetic modification technology provides a fast, simple, and reliable method for establishing liver cancer models. That makes this kind of models suitable for related gene function research. Genetically engineered liver cancer mice can provide ideal experimental models for exploring the pathogenesis of liver cancer, drug screening, and clinical medical research. However, similar to the induced models, mice will have multiple and scattered tumor sites in the liver. Therefore, it is not a great model to compare the effect of different drugs because it is difficult to identify whether the size of tumor is affected by the drug or mouse itself. The poor uniformity makes it unsuitable for pharmacological experiments. Thus, HCC transplantation mouse model is available.

## Hepatocellular Carcinoma Transplantation Mouse Models

The transplanted liver cancer mouse model refers to an animal model formed by transplanting mouse or human liver cancer tissues or cell lines into mice (76). This model is constructed fast, easy, and suitable for anticancer drug tests in preclinical trials. Also, human resource cells could be used in this kind of model, which makes a long-term development of the evaluation of liver cancer transplantation mouse model (77).

The early-stage transplantation model was mainly based on the transplantation with the same gene strain tumor cells on homologous mice. Transplanted H22 cells into BALB/C mice (78) or Hepa1-6 cells into C57 mice (79) subcutaneously or *in situ* could be constructed as a mouse resource HCC model. Subcutaneous tumors are easier to be observed and monitored, and *in situ* tumors mimic the local environment better (80). However, from a germline perspective, the difference gap between mice and human is still too big. Scientists then tried to transplant human liver cancer cells or tissue blocks into immunodeficient mice to build the human resource liver cancer model. A kind of mice with specific lack of certain immune cells was constructed, such as Rag1^-/-^, Rag2^-/-^, or nude mice. Rag1 and Rag2 are the key enzymes in the TCR and BCR VDJ recombination steps of T and B cells. Without these enzymes, mature T and B cells cannot be formed. That makes Rag1^-/-^ and Rag2^-/-^ mice have similar phenotypes that result in severe early developmental arrest of T/B cells. T cells arrested at the CD3^-^CD4^-^CD8^-^CD25^+^ stage, and B cells arrested at the B220^-^CD43^+^IgM^-^ progenitor B-cell stage. Their peripheral blood does not have mature circulating T/B lymphocytes, which is very similar to human severe combined immunodeficiency syndrome (SCID) (81). Nude mice, without thymus and hair, lack T lymphocytes and have decreased T-cell and B-cell function (82). These immunodeficient mice are broadly used to construct a carcinoma transplantation model. The first human HCC cell line, BEL-16, was established in 1963 (83). The technology of mouse HCC models that were constructed using human HCC cells becomes more and more mature. HepG2, Huh7, Hep3B, and other HCC cell lines succeeded in building the carcinoma transplantation models.

### Heterotopic Transplantation Mice

In the heterotopic transplantation method, planting the tumor subcutaneously such as at the back or underarm is the most common method (84). This method has been used in preclinical assessment of anti-liver cancer treatment because of the short period of tumor model construction and the convenience for detection. This method has a high tumor formation rate, short cycle, easy control of tumor size and location, small individual differences, similar effects on the host, and easy to objectively judge the efficacy, but it cannot mimic the tumor microenvironment (85).

### Orthotopic Transplantation Mice

Many studies reported that the microenvironment has a very important influence on the biological behavior of malignant cells (86). Compared with heterotopic transplantation, orthotopically (*in situ*) transplanted tumors can further reflect the true condition of the tumor and enhance the reliability of the tumor model (87). Many subcutaneously transplanted tumor cell lines do not spontaneously induce metastasis, and metastasis could be induced in orthotopic transplantation models. It indicates that tumor cells and organ-specific factors can interact with each other to play an important role in the occurrence of liver cancer (88). On the other hand, *in situ* transplantation can mimic the immune microenvironment of the tumor (82). Therefore, most results based on the ectopic model should be further verified by the orthotopic transplantation model. *In situ* growth can better simulate the microenvironment of tumor cells growing in the body, and the prediction of drug efficacy is more accurate (89). However, surgical transplant procedures are complicated and expensive. Also, it is difficult to quickly detect the tumor growth and tumors’ response to drugs.

It is important to choose the right kind of mice and inject the carcinoma cells in the right place. For the orthotopic transplantation model, there are two main ways: subserosal injection and surgical orthotopic transplantation. For the former one, the tumor cells should be directly injected into the subserosa (90). In order to prevent dispersing of cell into tissue, the effective method is to add Matrigel into the cell suspension to construct a single solid tumor (91). However, it sometimes still becomes multiple scattered tumors. Therefore, surgical orthotopic transplantation to bury a little tumor cube into the *in situ* tissue could be chosen. The size of implant tumor is usually about 1 mm^3^. They are from surgical excision of liver cancer patients or a subcutaneous mass of HCC (92).

### Patient-Derived Xenograft Mice

To better integrate clinical work, scientists began to use the patient-derived xenograft (PDX) model in order to maximally mimic the true microenvironment of the tumor (93). A cube dice of patient’s tumor tissue could be put into nude mouse liver directly. The nude mice have a defective T-cell immune system, which will lower the incidence rate of rejection (94). However, there are two problems for this model. Firstly, the success rate of PDX is less than 20% all over the world right now (95). Secondly, because nude mice have a defective T-cell immune system, this model is not good for immunological research (96). In addition, there are some ways to solve these problems. The tumor cube could be transplanted subcutaneously first in mice. When the tumor grew in mice, researchers can transplant the cube for heterotopic transplantation. After undergoing the adaptation process, the *in situ* tumor formation rate will greatly increase, but that will decrease the level of mimic for human patients (97). On the other hand, they could inject human serum and T cells into mice to rebuild the immune system (98, 99).

Although the *in situ* tumor is easy to form into a single tumor, the overall uniformity of the tumor mass is still poor, and the quality control method is not that well as heterotopic. To increase the uniformity of the tumor cube, it needs to make the surgical procedure of the orthotopic liver transplantation model a little more elaborate. Furthermore, the detection of tumor growth and drug response in the orthotopic liver transplantation model is not as easy as that of the heterotopic transplantation model. The predictive value of these models in the actual antitumor effect in patients is still unclear. All of these have severely limited the use and value of transplanted mouse models of liver cancer.

## Other Mouse Hepatocellular Carcinoma Models

### Hepatitis B Virus-Infected Mouse Model

HBV infection is an important cause of HCC. More than 80% of human liver cancer is caused by HBV infection (100). The most studied components of HBV are hepatitis B virus X protein (HBx) and hepatitis B virus surface antigen (101, 102). Previous studies have shown that the HBV outer membrane large protein, X protein, is the core factor of hepatocellular carcinogenesis (103). However, human HBV could not induce hepatitis in mouse hepatocytes. Therefore, the establishment of these animal models of HBV usually requires the insertion of human hepatocytes (104). In 1985, scientists integrated HBV DNA sequences into the genome of mice, constructing a transgenic mouse model of chronic carriers of HBV infection (105). The transgenic mice incorporating HBx gene showed progressive changes in liver pathology. However, it can trigger the immune tolerance of mice themselves (106). Therefore, immunodeficient mice are generally used to establish a corresponding liver cancer model. Subsequently, a large number of transgenic mouse models carrying the HBV genome and a single HBV gene initiated by the HBV promoter or liver-specific promoter were established. These models can be used to study liver injury and malignant transformation of cells *in vivo* and provide conclusive evidence that viral genes can initiate and promote the occurrence of HCC (107). Liver cells will be changed at 4 months age of mice. Adenomas will appear in 8–10-month-old mice. More than 80% of male mice with HCC will die at 11–15 months, and more than 60% of female mice died at 17–21 months. This mouse model provides a research method for studying the pathogenesis, treatment method, and drug screening against human hepatitis B and has important medical application value (107). Due to the relative unpredictability of such mouse models, the tumor formation model is poorly uniform. At the same time, limited by mouse strains, it has greatly hindered the promotion of HBV-infected mice as a research model of liver cancer.

### Hepatocellular Carcinoma Metastasis Model

In the process of tumor development, metastasis is a very important dividing line. When the tumor becomes metastasized, it means that the malignancy of the tumor is extremely high (108). Because there is almost no possibility for a complete section surgery after metastasis, the vast majority of tumor patients eventually die of multiple organ failure after metastasis. This is related to the overall prognosis and survival of the patient (109). Therefore, the study of metastasis is also an important part of cancer research. There are two common kinds of HCC metastasis model construct methods. The first one is liver cancer lung metastasis model (110). This is a mature method. What researchers need to do is inject the suitable cell lines into the tail vein of mice. Because all of the vein blood will go back to the heart through the lung, that makes this model stable (111). The other one is a method of liver cancer bone metastasis. HCCLM3 is a cell line established from pulmonary metastatic lesions produced in nude mice, which can be used as subcutaneous inoculation of the HCC cell line. We injected HCCLM3 into the heart of mice directly. The tumor cells will colonize into bone marrow with the flow of blood all over the body. When the tumor grows in the bone marrow, we removed the tumor and extracted the primary cells. The above process was repeated several times, and a pro-bone metastasis liver cancer cell line was obtained, which can make a high incidence of bone metastasis (112, 113). The metastasis models can help us further study the relevant mechanisms of tumor metastasis and develop targeted treatments in turn. However, in addition to lung metastasis models, various other metastasis models are currently immature and require continued research by scientists.

## How to Choose the Model Type

**Figure 2:**
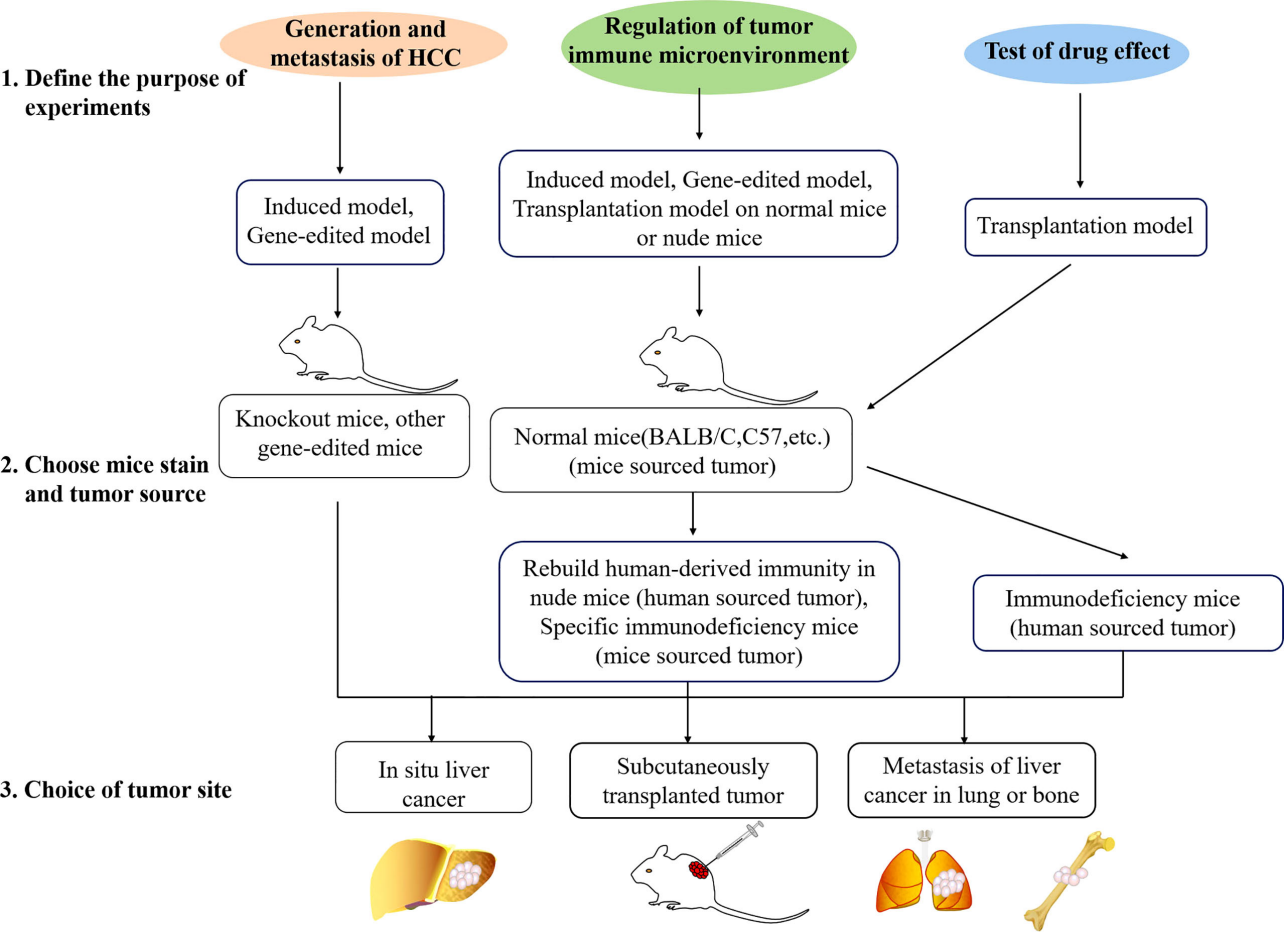
Three steps used to choose a mouse model. Firstly, the purpose of the experiment should be defined. Studies are usually about generation of HCC, immune microenvironment, or drug effect test. Different animal models are available for different experimental purposes. Secondly, the mouse strain and tumor source should be chosen. Depending on different purposes, gene-edited mice, SCID mice, normal mice, and mouse- or patient-derived tissue, human or mouse cell lines could be chosen. Lastly, researchers should choose the tumor site. Most of the time, the subcutaneous model will be chosen in the initial test, and orthotopic or metastasis model will be used in the final test.

When researchers start experiments, the problem that will often be faced is the choice of animal models. Three steps used to choose a mouse model are shown in **Figure 2**. The correct model selection is the premise to ensure the experiment to proceed smoothly and obtain the desired results. Before the model type is chosen, there are a few points that need to be clear.

### Define the Purpose of the Study

Each model has its own type of application. For a tumor killing experiment of pharmacodynamics test, tumor size is the most important character. The tumor formed in the model needs to be single tumor-forming and measurable. In this regard, subcutaneously transplanted tumor cell lines and *in situ* liver tumor tissue transplant are the best choices. For the observation of intrahepatic metastasis, injecting tumor cells into mouse liver directly might be a great choice. The induced or gene-edited mouse HCC model is fit for the research of occurrence and development of liver cancer or the study of the relationship of some genes or pathways. For the research of targeted metastasis of liver cancer, metastasis models could be chosen. Also, combining different kinds of models to make comparisons is a great choice for immunity research.

### Choice of Mouse Strain and Tumor Source

After making a decision of model type, the next step is to choose the mouse strain and tumor source. There are many conditions. For example, nude mice or some other immunodeficiency mice could be used for human-source experiments. Normal mice (BALB/C, C57BL/6, etc.) or rebuilding human-derived immunity in nude mice (to prevent rejection of a xenotransplantation) could be used for immunity-related experiments. Various mice could be used for related research on induction models. Knockout mice or other gene expression methods are the best way to study the role of a specific gene or the occurrence and development of liver cancer under the influence of pathways.

After the mice have been selected, which kind of tumor source also needs to be decided. For the cell line model, there are two types, human liver cancer cell lines and mouse liver cancer cell lines. Generally, for the convenience of experiments, researchers always choose subcutaneous tumor formation with cell lines at the initial experiments. At this time, HCC cell lines related to their own research, such as the expression level of the target gene and the background of liver cancer cells, will be considered. Under some spatial requests according to the experimental needs, cells can also be used after gene editing. Induced models and gene-edited models can be used for immune-related studies. Immunological normal mice and homologous mouse liver cancer cells for tumor formation modeling (such as H22-BALB/C; Hepa1-6-C57) are great choices too.

However, all of the cell lines have been immortalized, which means that they are different from the real situation and cannot reflect the tumor microenvironment well (114). At this time, most researchers hope to inoculate the tumor tissues from the patients directly into the mice in order to restore the traits of the tumor itself and the microenvironment maximum. This is the PDX model. But the nude mice used in the PDX model are thymus immunodeficient, which means they are T-cell immunodeficiency mice (115). Therefore, when doing immune-related research, the choice of this model must be particularly careful. One of the possible methods is to reinject human serum to reconstruct human immunity. In general, the purpose is to select the best tumor resource fit for the experimental purpose.

### Tumor Site

After deciding the mouse breed and tumor resource, the final selection is the tumor site. The tumor of the induced model or a gene-edited model, which are all original liver tumor, will be *in situ*. At the time frame of the experiment, the liver should be dissected out for observation and evaluation. It is generally recommended to transplant tumor tissue blocks of patients with liver cancer directly *in situ* of nude mice for PDX models (116, 117). However, in view of the low success rate, sometimes the tumor mass is first planted subcutaneously in nude mice. After the mice are tumor-bearing, the tumor mass could be moved from the subcutaneous and planted into the liver. This method can effectively improve the success rate, but it is relatively discounted in the direct credibility of the evidence. Generally, the initial test is to subcutaneously inject the cultured HCC cells directly into mice to form a skin mound. This method has the advantages of simple operation, high success rate, good uniformity, easy observation and measurement at any time, and the most practical planting site. However, in view of enhancing the credibility of the evidence or excluding the effects of special microenvironment, *in situ* cultivation sometimes needs to be performed. In addition, in the directional metastasis of liver cancer, tumors are selected to be formed in other specific organs (such as the lung or the bone). Through these three steps, a suitable HCC model could be decided.

## Summary

Because different etiologies cause changes in different patients, resulting in liver cancer heterogeneity, liver cancer models are not universal. Up to now, the most commonly used models are still chemically induced liver cancer models, knockout mouse naturally induced liver cancer models, and tumor-implanted mouse liver cancer models. These models have their own advantages and disadvantages, so they are adapted to different experimental requirements. The mouse liver cancer model of human-resourced cell line is regarded as the model closest to the real nature. However, due to the allogeneic rejection immune response in mice, this model could not play a role in immunological research. Although rebuilding the human immune system in mice can effectively solve this problem, it is also limited by cost, and this model cannot be popularized well. In addition, if we are talking about the cutting-edge model, it is the PDX model and the organoid model (118, 119). They both use patient-derived tumor tissues and hope to use mice or culture medium as the basic environment to cultivate these tumor tissues to better simulate the real environment of tumors. Recent studies indicated that the genetically modified liver organoids by either RNA interference or CRISPR/Cas9 technology can induce liver cancers that display characteristics of human CCA and HCC in immunocompetent mice (120). Both models are highly simulated and convincing. However, limited by experimental techniques, the success rates of both models are now still very low. In addition, the cost of modeling is quite high. This also directly leads to the temporary inability to widely promote the use of these two models. It is believed that with the development of technology, the maturation of primary immune cells *in vitro*, and the technology of immunosuppression, the application of these two models will be greatly increased.

## Author Contributions

Conceptualization: FH and LC. Writing: SL, FH, GR, YW, BZ, XC, and LC. Supervision: YW and LC. Project administration and funding acquisition: FH and LC. All authors have read and agreed to the published version of the article.

## Funding

This work was supported by grants from the National Natural Science Foundation of China (Nos. 82172971, 31671348, and 31301064), the Public Welfare Technology Project of Zhejiang Province (LGF21H160033), the Zhejiang Medical Technology Plan Project (2021KY047), and the Grant for 521 talent project of ZSTU, Chen Xiao-ping Foundation for the Development of Science and Technology of Hubei Province (CXPJJH12000001-2020317).

## Conflict of Interest

The authors declare that the research was conducted in the absence of any commercial or financial relationships that could be construed as a potential conflict of interest.

## Publisher’s Note

All claims expressed in this article are solely those of the authors and do not necessarily represent those of their affiliated organizations, or those of the publisher, the editors and the reviewers. Any product that may be evaluated in this article, or claim that may be made by its manufacturer, is not guaranteed or endorsed by the publisher.
